# Vibrotactile somatosensory stimulus to assist the transition from level walking to stair ascent in the elderly: a pilot study

**DOI:** 10.1186/s12891-022-05093-3

**Published:** 2022-02-24

**Authors:** Kiyoung Kwak, Seunghun Ko, Dongwook Kim

**Affiliations:** 1 Division of Biomedical Engineering, Jeonbuk National University, 567 Baekje-daero, Deokjin-gu, Jeonju-si, Jeollabuk-do Republic of Korea; 2 Department of Healthcare Engineering, Jeonbuk National University, 567 Baekje-daero, Deokjin-gu, Jeonju-si, Jeollabuk-do Republic of Korea; 3 Research center for Healthcare & Welfare Instrument for the Elderly, Jeonbuk National University, 567 Baekje-daero, Deokjin-gu, Jeonju-si, Jeollabuk-do Republic of Korea

**Keywords:** Stair ascent, Somatosensory, Vibration, Elderly, Biomechanics, Level walking

## Abstract

**Background:**

Although, in daily living, almost all stair ambulation is conducted posterior to level walking, or vice versa, there are only a few studies related to the transition compared to the studies on steady-state stair walking.

Furthermore, neuromotor control in the instant of the transition is different from that of the steady-state stair walking. However, there are only a few studies investigating the transition from level walking to stair ascent in the elderly by comparing with young adults, and there is no study on the assistance of the transition movement in the elderly who are experiencing neurophysiological changes.

Thus, this pilot study aimed to compare the flat surface-to-stair ascent transition by the elderly to that seen in young adults, and to investigate how vibrotactile somatosensory stimulus (VSS), which has a positive effect on muscle performance and gait, affects the transition tasks in elderly people.

**Results:**

In the first half of the stance phase, the elderly exhibited a higher moment and power of the hip extensor and a less moment and power of the knee extensor compared with young adults. In the second half of the stance phase, positive plantar-flexor power and support moment was higher in the elderly. In addition, during the single-limb support phase, dorsiflexion was maintained in the elderly, whereas young adults appeared to have decreased dorsiflexion.

When the VSS was applied, the moment and power of the hip extensor, the plantar-flexor moment, and the support moment in the entire of the stance phase were increased. In addition, it was found that the degree of the kinetics parameters was different depending on the frequencies of the VSS.

**Conclusions:**

This pilot study has revealed evident biomechanical differences between elderly people and young adults during the transition from level walking to stair ascent. Additionally, it has shown that the VSS may accentuate the features of the transition movement of the elderly and regulate joint kinetics. The results of the present pilot study can provide a base for further research and understanding of movement, which can be utilized in designing assistance aids for the elderly.

**Trial registration:**

CRIS, KCT0005434, Registered 25 September 2020, Retrospectively registered.

## Background

Ascending and descending the stairs is a more complicated movement than level walking, given the effect of gravity on the body and the specifications of the stairs. Hence, stair ambulation will be a challenging task for the elderly who experience neurophysiological changes, causing a decrease in muscle strength, reaction speed, and cognitive processing ability. For these reasons, stair negotiation by the elderly has been investigated by numerous studies and well documented [1–12].

In daily living, stair walking is mostly performed before or after level walking. However, participants in previous studies were asked to initiate walking the stairs from a static upright posture in front of the staircase. That is, former studies did not consider a realistic stair walking performed in daily living. In addition, most studies have focused on steady-state stair ascent and descent. Actually, we must face an instant of the transition from flat ground walking to stair ascent or descent.

It was reported that the transition requires adjustments in control of motion before the subjects set into a steady-state that continues over the following steps [7]. In other words, there will be another difference because the transition from level walking to stair walking, or vice versa, is accompanied by task recognition, gait strategy design for successful transition, and changes in movement modality. But, the transition is not fully investigated, while the steady-state stair walking has been much examined and well understood. Further, there are few studies to assist stair walking of the elderly.

There are a few studies investigated the transition from level walking to stair walking. Vallabhajosula et al. [13, 14] investigated the transition from level walking to stair climbing, and they reported the differences in joint kinetics (i.e., moment and power) between transition and steady state stair climb. However, the participants of their study were only young adults. Singhal et al. [15] investigated joint kinetics at the instant of transition from walking on flat ground to going down stairs for the elderly, but their results were about gender differences. Alcock et al. [16] reported that biomechanical differences between the 2-step transitioning and continuous stair ascent in older women. Carli et al. [17] compared only ground reaction force during the floor-to-stair transition gait in the elderly.

Although the transition movements are being investigated, the number of studies is limited compared to studies investigating steady-state stair ambulation. Moreover, there are no studies that have revealed the characteristics of the elderly by comparing it to that of young adults. To assist stair walking in the elderly who experience neurophysiological changes, it is important to reveal their characteristics in comparison with young adults. In addition, as mentioned earlier, the elderly experience neurophysiological weakness due to aging, and stair walking is a complex task. Therefore, a proof of concept study related to the support of stair walking for the elderly is needed. Hence, it is important to apply a means that can support and investigate its effect.

In this study, the transition from level walking to stair ascent was targeted, and vibrotactile somatosensory stimulus (VSS) was applied as a means of supporting during the transition movement. The VSS has been found to have various advantages through many studies. The VSS is the applying mechanical vibration to initially relaxed skeletal muscle or its tendon [18]. It was reported that involuntary enhancement of electromyogram (EMG) and muscle contraction strength is induced when mechanical vibration is applied [19]. Based on those results, positive influence of the VSS on muscle performance, strength, and power [20–23], effects on gait control [24, 25], and implications for rehabilitation [26] had been investigated. Therefore, the VSS could be utilized as a means to assist the transition from level walking to stair ascent of the elderly.

Thus, the present pilot study aimed to reveal the characteristics of level walking-to-stair ascent transition in the elderly by comparing it to young adults, and to investigate the effects of the VSS on the transition movement, and to discuss the VSS as a means to assist the transition movement.

## Methods

### Participants

To investigate the characteristics of transition movement in the elderly, young adults and older adults over 65 aged were recruited. A total of fifteen young adults (age: 25.5 ± 1.5 years old, height: 173.2 ± 2.6 cm, weight: 72.4 ± 4.4 kg) and ten elderly (age: 76.0 ± 1.7 years old, height: 166.7 ± 3.7 cm, weight: 68.0 ± 5.5 kg) participated in the study. Participants in the present study are the same as those in Peter et al., our previous study [27]. All participants had no musculoskeletal diseases, and were free from any neuro-physiological diseases, and could perform the level walking and stair climbing without any assistance. All participants provided written informed consent prior to conducting and participating in this study. The present study was approved by the Institutional Review Board of Jeonbuk National University (IRB File No. JBNU 2017–03–011-001).

### Vibrotactile somatosensory stimulus

**Fig. 1:**
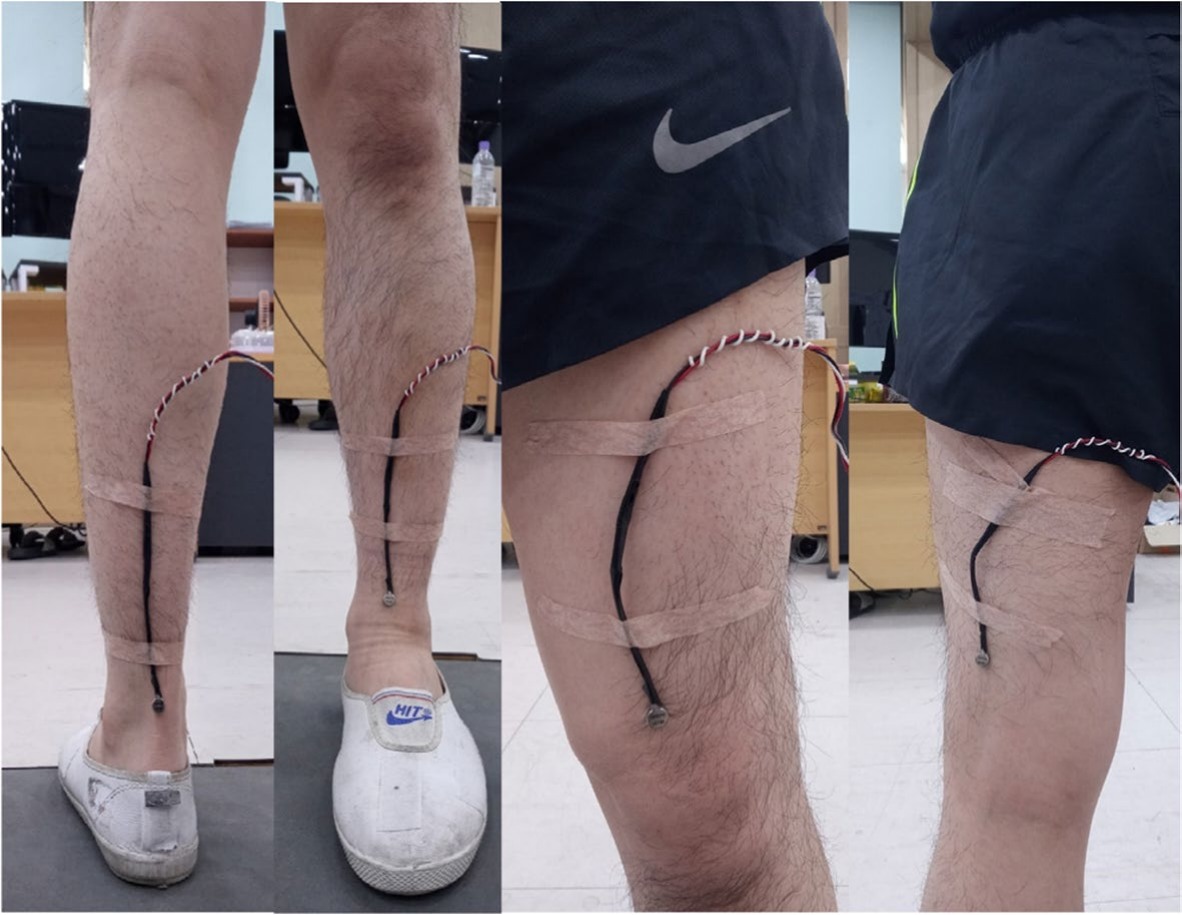
Example of linear actuators attachment

To apply the VSS during the transition movement, small linear actuators (DMJBRN0934AA, Samsung Electro-Mechanics Co., Ltd., Korea) were attached to four targeted sites as follows: tibialis tendon, Achilles tendon, quadriceps femoris tendon, and biceps femoris tendon as shown in Fig. 1.

To regulate the frequency and intensity of the VSS, a function generator (AFG-2125, Good Will Instrument Co., Ltd., Taiwan) was used. In our previous studies [28, 29], the vibrotactile perception threshold in the range of 100 Hz to 300 Hz, where the skin mechanoreceptor is sensitive [30], was measured. As a result, 190 Hz was most sensitive, 180 Hz was similar to 190 Hz, and perception threshold rapidly increased at 200 Hz. Based on these results, frequencies of 180 Hz, 190 Hz, and 250 Hz were used in the present study so that the VSS conditions consisted of non-stimulus (NS, i.e., without the VSS), 180 Hz, 190 Hz, and 250 Hz.

The VSS intensity was 80% of the vibrotactile perception threshold. It has been reported that the response of the central nervous system appears below the perception threshold level [31, 32], and that intensity upper perception threshold can cause discomfort or instability of postural balance [33, 34].

### Materials and instrumentation

**Fig. 2:**
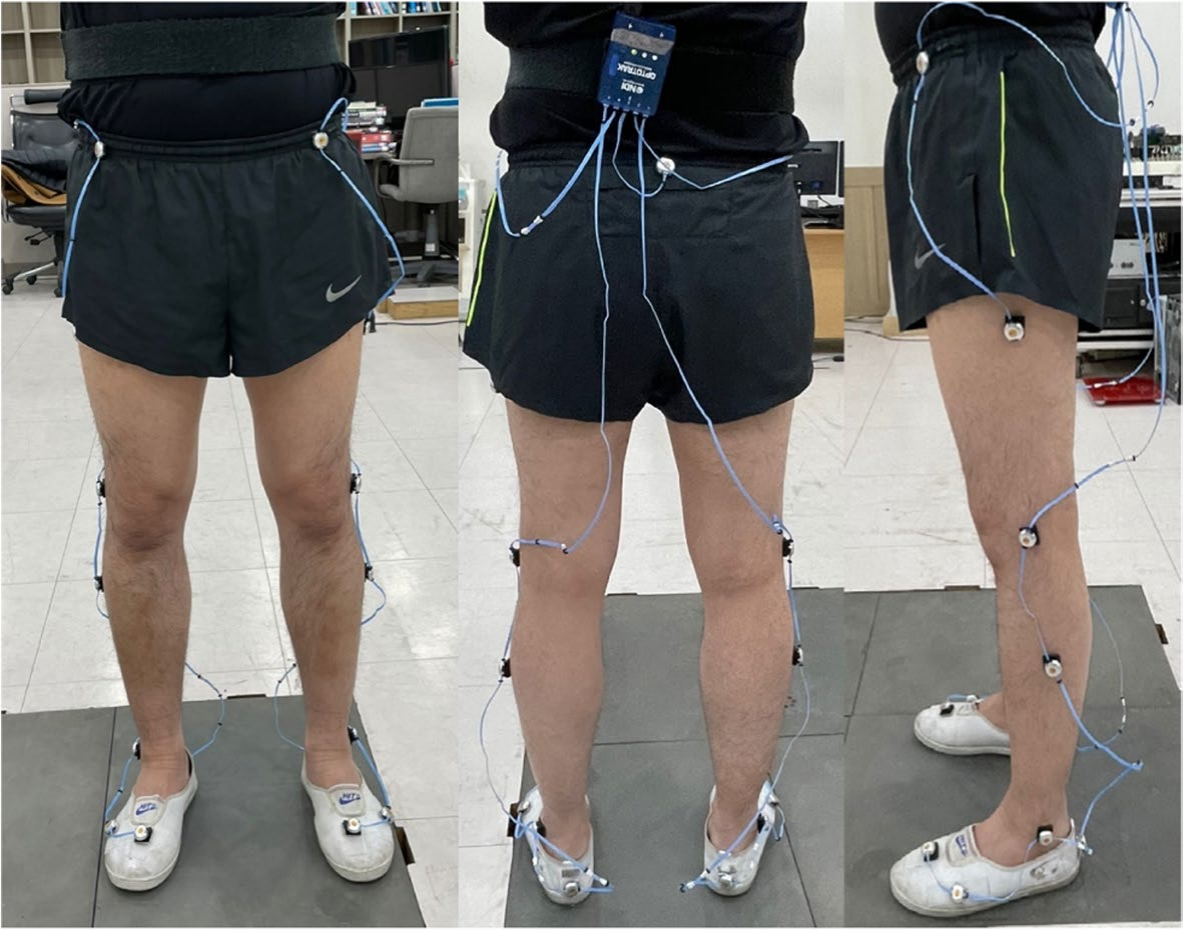
Placement of infrared light-emitting diodes

To capture the transition movement, a 3-D marker-based motion capture system, which is considered the standard method of movement analysis [35], was used. Infrared light-emitting diodes (Smart marker, Northern Digital Inc., Canada) were placed according to the motion module marker guide (MusculoGraphics, Inc., USA) as shown in Fig. 2.

**Fig. 3:**
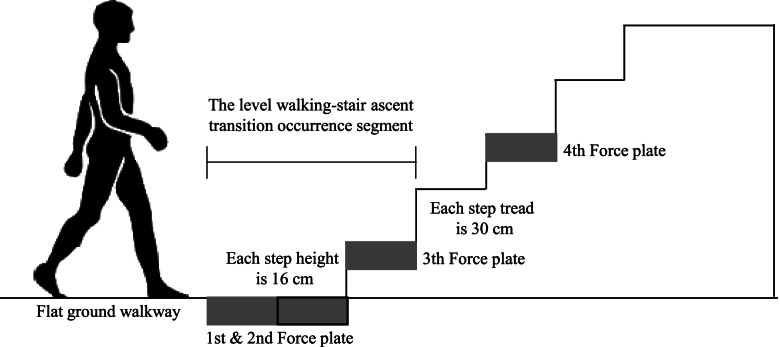
Custom-built wooden staircase used in the present study

A total of three position sensors (Optotrak Certus, Northern Digital Inc., Canada) and four force plates (Bertec Corp., USA) were used to record participants’ movement and ground reaction force. Custom-built wooden staircase, as shown in Fig. 3, was used.

### Procedure

All participants walked 3 m on a flat ground walkway at a self-selected speed before stair climbing, and then they continued to perform stair climbing without stopping. They completed a given task three times for each VSS condition. All the VSS conditions were applied randomly during the given task.

### Data analysis

All kinematics (i.e. joint angle) and kinetics (i.e. joint moment and joint power) of the lower extremity joints (i.e. hip, knee, and ankle) were calculated using the software for Interactive Musculoskeletal Modeling (SIMM, MusculoGraphics Inc., USA), and support moment was calculated according to Winter [36]. Joint moment, support moment, and joint power were normalized to the participants’ body weight, and the duration from the toe-off to the toe-off of the ipsilateral leg was normalized from 0 to 100%.

Profiles of joint angle, moment, and power for each participant derived during the three trials for each VSS condition were ensemble-averaged. Then, to derive a grand ensemble for the VSS conditions, ensemble-averaged profiles were ensemble averaged.

**Fig. 4:**
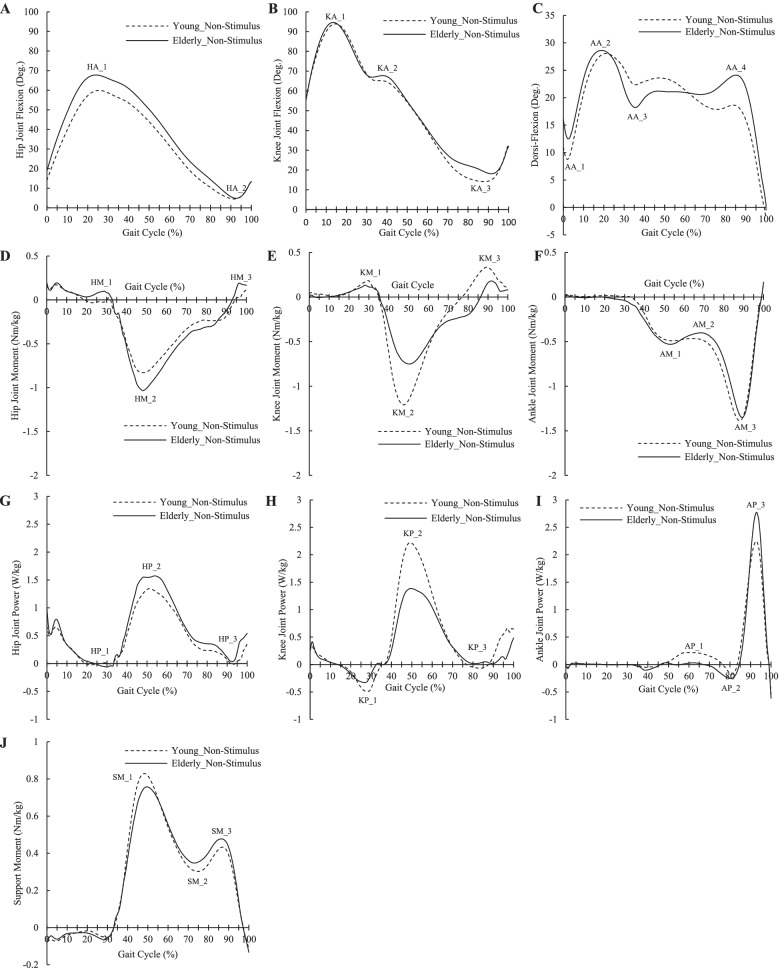
Profiles of lower-limb joints in both groups during transition from level walking to stair ascent. **A** Hip joint angle; **B** Knee joint angle; **C** Ankle joint angle; **D** Hip joint moment; **E** Knee joint moment; **F** Ankle joint moment; **G** Hip joint power; **H** Knee joint power; **I** Ankle joint power; **J** Support moment; For the joint angle, (+) indicates joint flexion and (−) indicates joint extension; For the joint moment, (+) indicates joint flexor moment and (−) indicates joint extensor moment; For the support moment, (+) indicates sum of extensor moments of the lower-limb joints; HA: Hip joint angle; KA: Knee joint angle; AA: Ankle joint angle; HM: Hip joint moment; KM: Knee joint Moment; AM: Ankle joint moment; HP: Hip joint power; KP: Knee joint power; AP: Ankle joint power; SM: Support moment

**Fig. 5:**
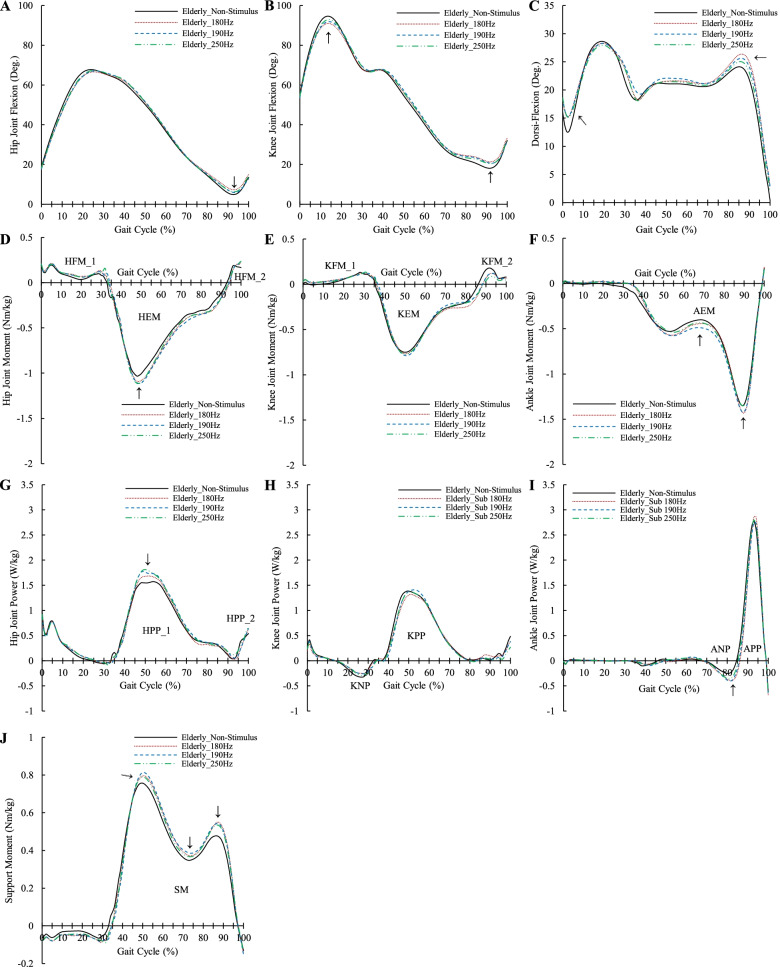
Profiles of the elderly during transition from level walking to stair ascent upon VSS application. **A** Hip joint angle; **B** Knee joint angle; **C** Ankle joint angle; **D** Hip joint moment; **E** Knee joint moment; **F** Ankle joint moment; **G** Hip joint power; **H** Knee joint power; **I** Ankle joint power; **J** Support moment; For the joint angle, (+) indicates joint flexion and (−) indicates joint extension; For the joint moment, (+) indicates joint flexor moment and (−) indicates joint extensor moment; For the support moment, (+) indicates sum of extensor moments of the lower-limb joints; Arrows represent change by the VSS; HFM: Hip flexor moment; HEM: Hip extensor moment; KFM: Knee flexor moment; KEM: Knee extensor moment; AEM: Ankle extensor moment; HPP: Hip positive power; KNP: Knee negative power; KPP: Knee positive power; ANP: Ankle negative power; APP: Ankle positive power; SM: Support moment

To examine the features of the transition movement of the elders, ensemble-averaged profiles are shown in Fig. 4. In addition, grand ensemble-averaged profiles are presented in Fig. 5 to investigate the effects of the VSS on the transition from level walking to stair ascent in the elderly. Peak joint angles, means of joint moment, support moment, and joint power in a specific period are presented in Tables 1, 2, 3 and 4 to determine the effects of the frequency of the VSS.

**Table 1:** Peak joint angle parameters depending on the frequency of the VSS in the elderly

		Elderly Non-Stimulus	Elderly 180 Hz Stimulus	Elderly 190 Hz Stimulus	Elderly 250 Hz Stimulus
Hip Joint Angle	HA_1	67.8 ± 4.1	66.6 ± 3.8	67.2 ± 3.8	67.4 ± 3.3
	95% CI		[− 0.26, 2.64]	[−0.82, 2.07]	[− 0.88, 1.74]
	t-value*		1.85	0.98	0.74
	*p* value*		0.098	0.353	0.480
	HA_2	4.9 ± 5.1	7.3 ± 3.5	5.9 ± 4.8	6.3 ± 5.1
	95% CI		[−5.81, 1.05]	[−2.41, 0.39]	[−3.28, 0.39]
	t-value*		−1.57	−1.63	− 1.79
	*p* value*		0.151	0.137	0.108
Knee Joint Angle	KA_1	94.6 ± 4.3	*91.0 ± 7.1	*92.0 ± 8.5	92.9 ± 8.5
	95% CI		[0.89, 6.34]	[0.88, 5.34]	[−1.21, 4.70]
	t-value*		3.022	2.34	1.34
	*p* value*		0.015	0.044	0.214
	KA_2	68.1 ± 2.9	68.3 ± 2.2	69.6 ± 3.3	69.3 ± 3.6
	KA_3	18.1 ± 5.4	*21.3 ± 4.6	*20.6 ± 5.6	*20.4 ± 5.7
	95% CI		[−5.33, −1.06]	[−4.28, −0.71]	[−4.19, −0.32]
	t-value*		−3.39	−3.16	−2.64
	*p* value*		0.008	0.012	0.027
Ankle Joint Angle	AA_1	12.6 ± 3.5	*15.3 ± 3.4	*15.4 ± 3.7	*15.2 ± 3.6
	95% CI		[−4.62, −0.75]	[−5.51, − 0.02]	[− 5.11, − 0.03]
	t-value*		−3.14	−2.28	− 2.29
	*p* value*		0.012	0.049	0.048
	AA_2	28.6 ± 2.8	28.3 ± 3.2	28.3 ± 3.1	28.0 ± 2.8
	AA_3	18.2 ± 4.0	18.3 ± 5.1	19.3 ± 4.7^c^	18.1 ± 5.3^b^
	95% CI^a,b,c^		–	[0.03, 2.30]	[0.03, 2.30]
	t-value^a,b,c^		–	2.32	2.32
	*p* value^a,b,c^		–	0.045^c^	0.045^b^
	AA_4	24.1 ± 3.2	*26.4 ± 2.4	*25.6 ± 2.4	25.0 ± 2.4^a^
	95% CI*		[−3.97, −0.50]	[2.95, −0.04]	[−2.82, 0.97]
	t-value*		−2.91	−2.33	−1.10
	*p* value*		0.017	0.045	0.298
	95% CI^a,b,c^		[0.58, 2.03]	–	[0.58, 2.03]
	t-value^a,b,c^		4.07	–	4.07
	*p* value^a,b,c^		0.003^c^	–	0.003^a^

Mean ± Standard deviation, Unit: Degree, *CI* Confidence interval, *t-value* paired t-test

^*^ indicates significant difference between Non-Stimulus and Stimulus (*p* < 0.05)

^a^ indicates significant difference from 180 Hz stimulus (*p* < 0.05)

^b^ indicates significant difference from 190 Hz stimulus (*p* < 0.05)

^c^ indicates significant difference from 250 Hz stimulus (*p* < 0.05)

**Table 2:** Joint moments depending on the frequency of the VSS in the elderly

		Elderly Non-Stimulus	Elderly 180 Hz Stimulus	Elderly 190 Hz Stimulus	Elderly 250 Hz Stimulus
Hip Joint Moment	HFM_1	0.093 ± 0.048	*0.114 ± 0.045^bc^	*0.109 ± 0.045 ^a^	*0.111 ± 0.043^a^
	Z-value*		−5.012	−5.012	− 5.012
	*p* value*		*p* < 0.001	*p* < 0.001	*p* < 0.001
	Z-value^a,b,c^		–	−3.855	−2.676
	*p* value^a,b,c^		–	*p* < 0.001^a^	0.007^a^
	HEM	−0.513 ± 0.299	*-0.544 ± 0.317	*-0.560 ± 0.322	*-0.549 ± 0.318
	Z-value*		−6.331	−5.632	−5.897
	*p* value*		*p* < 0.001	*p* < 0.001	*p* < 0.001
	HFM_2	0.134 ± 0.069	*0.167 ± 0.064	*0.174 ± 0.063	*0.153 ± 0.078^ab^
	Z-value*		−2.201	−2.201	−2.197
	*p* value*		0.028	0.028	0.028
	Z-value^a,b,c^		−2.201	−2.201	–
	*p* value^a,b,c^		0.016^c^	0.004^c^	–
Knee Joint Moment	KFM_1	0.061 ± 0.043	0.060 ± 0.035	0.061 ± 0.035^c^	0.058 ± 0.035^b^
	Z-value^a,b,c^		–	−2.150	−2.150
	*p* value^a,b,c^		–	0.032^c^	0.032^b^
	KEM	−0.406 ± 0.218	*-0.384 ± 0.243^bc^	*-0.397 ± 0.230^ac^	−0.404 ± 0.225^ab^
	Z-value*		−4.462	−2.659	−1.575
	*p* value*		*p* < 0.001	0.008	0.115
	Z-value^a,b,c^		−4.544	−3.861	− 4.991
	*p* value^a,b,c^		*p* < 0.001^b^	*p* < 0.001^c^	*p* < 0.001^a^
	KFM_2	0.111 ± 0.048	*0.080 ± 0.029^bc^	*0.083 ± 0.028^ac^	*0.055 ± 0.023^ab^
	95% CI		[0.02, 0.05]	[0.02, 0.05]	[0.04, 0.08]
	t-value*		4.49	4.15	6.49
	*p* value*		0.001	0.002	*p* < 0.001
	95% CIa,b,c		[−0.004, − 0.001]	[0.02, 0.03]	[0.02, 0.03]
	t-valuea,b,c		−3.81	11.92	10.49
	*p* value^a,b,c^		0.003^b^	*p* < 0.001^c^	*p* < 0.001^a^
Ankle Joint Moment	AEM	−0.465 ± 0.361	*-0.568 ± 0.359^bc^	*-0.597 ± 0.361^ac^	*-0.553 ± 0.351^ab^
	Z-value*		−4.598	−5.261	−4.454
	*p* value*		*p* < 0.001	*p* < 0.001	*p* < 0.001
	Z-value^a,b,c^		−3.689	−4.302	− 3.486
	*p* value^a,b,c^		0.015^b^	*p* < 0.001^c^	*p* < 0.001^a^

Mean ± Standard deviation, Unit: N∙m/kg (Newton meters/kilogram), +: Flexor moment, −: Extensor moment

*CI* Confidence interval, *t-value* paired t-test, *Z-value*Wilcoxon signed rank test

* indicates significant difference between Non-Stimulus and Stimulus (*p* < 0.05)

^a^ indicates significant difference from 180 Hz stimulus (*p* < 0.05)

^b^ indicates significant difference from 190 Hz stimulus (*p* < 0.05)

^c^ indicates significant difference from 250 Hz stimulus (*p* < 0.05)

**Table 3:** Support moment parameters depending on the frequency of the VSS in the elderly

		Elderly Non-Stimulus	Elderly 180 Hz Stimulus	Elderly 190 Hz Stimulus	Elderly 250 Hz Stimulus
Support Moment	SM	0.446 ± 0.189	*0.481 ± 0.195^bc^	*0.484 ± 0.202^ac^	*0.473 ± 0.195^ab^
	Z-value*		−5.785	−5.847	− 5.354
	*p* value*		*p* < 0.001	*p* < 0.001	*p* < 0.001
	Z-value^a,b,c^		−2.643	−4.916	−5.696
	*p* value^a,b,c^		0.008^b^	*p* < 0.001^c^	*p* < 0.001^a^

Mean ± Standard deviation, Unit: N∙m/kg (Newton meters/kilogram)

*Z-value* Wilcoxon signed rank test

^*^ indicates significant difference between Non-Stimulus and Stimulus (*p* < 0.05)

^a^ indicates significant difference from 180 Hz stimulus (*p* < 0.05)

^b^ indicates significant difference from 190 Hz stimulus (*p* < 0.05)

^c^ indicates significant difference from 250 Hz stimulus (*p* < 0.05)

**Table 4:** Joint power parameters depending on the frequency of the VSS in the elderly

		Elderly Non-Stimulus	Elderly 180 Hz Stimulus	Elderly 190 Hz Stimulus	Elderly 250 Hz Stimulus
Hip Joint Power	HPP_1	0.787 ± 0.532	0.786 ± 0.584^bc^	*0.851 ± 0.595^a^	*0.837 ± 0.604^a^
	Z-value*		−1.525	−5.782	−4.846
	*p* value*		0.127	*p* < 0.001	*p* < 0.001
	Z-value^a,b,c^		−4.710	−0.868	−6.463
	*p* value^a,b,c^		*p* < 0.001^b^	0.385^c^	*p* < 0.001^a^
	HPP_2	0.326 ± 0.193	0.323 ± 0.225^c^	0.328 ± 0.219^c^	*0.395 ± 0.195^ab^
	95% CI*		[−0.08, 0.03]	[− 0.09, 0.02]	[− 0.15, − 0.05]
	t-value*		−1.23	−1.36	−4.91
	*p* value*		0.264	0.222	0.003
	95% CI^a,b,c^		[−0.02, 0.01]	[−0.09, − 0.04]	[−0.11, − 0.04]
	t-value^a,b,c^		−0.75	−6.36	−4.93
	*p* value^a,b,c^		0.480^b^	0.001^c^	0.003^a^
Knee Joint Power	KNP	−0.170 ± 0.119	*-0.141 ± 0.091^c^	*-0.141 ± 0.092^c^	−0.154 ± 0.102^ab^
	Z-value*		−2.688	−2.427	−1.344
	*p* value*		0.007	0.015	0.179
	Z-value^a,b,c^		−1.792	−2.728	−3.584
	*p* value^a,b,c^		0.073^b^	0.006^c^	*p* < 0.001^a^
	KPP	0.689 ± 0.508	0.609 ± 0.483	*0.692 ± 0.517	*0.705 ± 0.489
	Z-value*		−0.198	−3.876	− 3.832
	*p* value*		0.843	*p* < 0.001	*p* < 0.001
Ankle Joint Power	ANP	−0.135 ± 0.090	*-0.214 ± 0.138	*-0.222 ± 0.137^c^	*-0.197 ± 0.126^b^
	95% CI*		[0.05, 0.13]	[0.06, 0.11]	[0.04, 0.08]
	t-value*		4.90	7.42	6.15
	*p* value*		*p* < 0.001	*p* < 0.001	*p* < 0.001
	95% CI^a,b,c^		–	[−0.04, −0.01]	[−0.04, − 0.01]
	t-value^a,b,c^		–	−3.35	−3.35
	*p* value^a,b,c^		–	0.004^c^	0.004^b^
	APP	1.510 ± 0.952	1.519 ± 1.021	1.410 ± 1.005^c^	1.598 ± 0.934^b^
	95% CI^a,b,c^		–	[−0.14, −0.02]	[−0.14, − 0.02]
	t-value^a,b,c^		–	−2.70	−2.70
	*p* value^a,b,c^		–	0.019^c^	0.019^b^

Mean ± Standard deviation, Unit: W/kg (Watt/kilogram), *CI* Confidence interval

*t-value* paired t-test, *Z-value* Wilcoxon signed rank test

^*^ indicates significant difference between Non-Stimulus and Stimulus (*p* < 0.05)

^a^ indicates significant difference from 180 Hz stimulus (*p* < 0.05)

^b^ indicates significant difference from 190 Hz stimulus (*p* < 0.05)

^c^ indicates significant difference from 250 Hz stimulus (*p* < 0.05)

### Statistical analysis

Peak joint angles, means of joint moment, support moment, and joint power were tested for normality using the Shapiro-Wilk test. Paired t-test and Wilcoxon signed rank test were used to test the effect difference between each frequency of the VSS. The statistical significance level was set at α < 0.05. Statistical analyses were conducted using SPSS Statistics version 20 (IBM, USA).

## Results

### Kinematics and kinetics in the young adults and the elderly during transitioning from flat ground to stair ascent

Joint profiles of biomechanics in the young vs. elderly males during transition from level walking-to-stair ascent are illustrated in Fig. 4.

In the early swing phase, the elderly showed greater dorsiflexion (AA_1) than that of the young, followed by greater hip joint flexion (HA_1). In the terminal swing phase, more extension of the ankle joint appeared in the elderly males.

Distinct differences in joint biomechanics of the two groups were observed during the stance phase (after 33% of the gait cycle). During the stance phase, the elderly showed greater flexion in the hip joint compared to the young adults. For the ankle joint, particularly, constantly decreasing dorsiflexion appeared in young adults; however, in the elderly, dorsiflexion was first sustained and then increased, followed by a rapid decrease. Compared with the young group, larger hip extensor moment and larger hip joint positive power, smaller knee extensor moment and smaller knee joint positive power, and larger peak plantar-flexor positive power appeared in the elderly. Similar results were also obtained for the support moment. Peak support moment was small in the first half of the stance phase, whereas a large support moment appeared in the second half of the stance phase.

### Changes in the joint profiles when VSS was applied

When the VSS was applied, joint biomechanics were affected, as shown in Fig. 5. For the joint angle, knee flexion was decreased and dorsiflexion was increased in the swing phase, whereas both knee flexion and dorsiflexion were increased in the stance phase. For the joint moment, an increase in hip extensor moment at the early stance phase then an increase in the plantar-flexor moment and a reduction in knee flexor moment were observed. For the joint power, both hip extensor positive power and plantar-flexor negative power were increased. Support moment was increased in all stance phases.

### Changes dependent on the frequency of the VSS

Significant changes depending on the frequency of the VSS are presented in Tables 1, 2, 3 and 4.

HA_1 without applying the VSS was 67.8 ± 4.1 deg. When the VSS was applied, HA_1 at 180 Hz, 190 Hz, and 250 Hz was 66.6 ± 3.8 deg., 67.2 ± 3.8 deg., and 67.4 ± 3.3 deg., respectively, with no differences between frequencies (all *p* > 0.05). Likewise, the second peak hip flexion angles (HA_2) for the NS condition, 180 Hz, 190 Hz, and 250 Hz were 4.9 ± 5.1 deg., 7.3 ± 3.5 deg., 5.9 ± 4.8 deg., and 6.3 ± 5.1 deg., respectively. There were no substantial differences between frequencies (all *p* > 0.05).

The first peak angle of the knee joint (KA_1) decreased when the VSS was applied except in the case of 250 Hz. KA_1 during the NS was 94.6 ± 4.3 deg., and in the case of 180 Hz, and 190 Hz stimulation was 91.0 ± 7.1 deg. (95% CI: 0.89–6.34, *p* = 0.015), and 92.0 ± 8.5 deg. (95% CI: 0.88–5.34, *p* = 0.044), respectively. The last flexion angle of the knee joint (KA_3) was 18.1 ± 5.4 deg. without the VSS. KA_3 increased significantly, when the VSS was applied. KA_3 was 21.3 ± 4.6 deg. at the 180 Hz (95% CI: − 5.33- -1.06, *p* = 0.008), 20.6 ± 5.6 deg. at the 190 Hz (95% CI: − 4.28- -0.71, *p* = 0.012), and 20.4 ± 5.7 deg. at the 250 Hz (95% CI: − 4.19- -0.32, *p* = 0.027).

AA_1 was 15.3 ± 3.4 deg. for the 180 Hz condition, 15.4 ± 3.7 deg. for the 190 Hz, and 15.2 ± 3.6 deg. for the 250 Hz, which was significantly greater than that of the NS condition (12.6 ± 3.5 deg., all *p* < 0.05). However, there was no significant difference between frequencies (all *p* > 0.05). The last ankle dorsiflexion peak (AA_4) of the NS was 24.1 ± 3.2 deg.. When the VSS was applied, AA_4 increased except at the 250 Hz stimulation. In the case of 180 Hz, and 190 Hz, AA_4 was 26.4 ± 2.4 (*p* = 0.017), and 25.6 ± 2.4 (*p* = 0.045), respectively. In particular, there was a significant difference between the 180 Hz and the 250 Hz conditions (180 Hz: 26.4 ± 2.4 vs. 250 Hz: 25.0 ± 2.4, *p* = 0.003).

Concerning the joint moment, the HFM_1 increased in all the VSS conditions. The HFM_1 without the VSS was 0.093 ± 0.048 Nm/kg. In the case of 180 Hz, 190 Hz, and 250 Hz stimulus, it was 0.114 ± 0.045 Nm/kg, 0.109 ± 0.045 Nm/kg, and 0.111 ± 0.043 Nm/kg (all *p* < 0.001), respectively. In addition, significant differences were observed between 180 Hz stimulus and both 190 Hz (Z-value: − 3.855, *p* < 0.001) and 250 Hz (Z-value: − 2.676, *p* = 0.007) stimuli.

Similarly, the HFM_2 increased when the VSS was applied. The HFM_2 during the NS condition was 0.134 ± 0.069 Nm/kg, and in the case of 180 Hz, 190 Hz, and 250 Hz stimulus was 0.167 ± 0.064 Nm/kg, 0.174 ± 0.063 Nm/kg, and 0.153 ± 0.078, respectively (all *p* < 0.05). Additionally, significant differences were found between 250 Hz stimulus and both 180 Hz (Z-value: − 2.201, *p* = 0.016) and 190 Hz (Z-value: − 2.201, *p* = 0.004) stimuli.

The mean moment of the hip extensor (HEM) was − 0.513 ± 0.299 Nm/kg in the case of NS. When the VSS was applied, HEM increased significantly in comparison to the NS. HEM was − 0.544 ± 0.317 Nm/kg at 180 Hz (Z-value − 6.331, *p* < 0.001), − 0.560 ± 0.322 Nm/kg at 190 Hz (Z-value: − 5.632, *p* < 0.001), and − 0.549 ± 0.318 Nm/kg at 250 Hz (Z-value: − 5.897, *p* < 0.001) stimulus. Significant differences between frequencies did not appear (all *p* > 0.05).

KEM decreased significantly during VSS application compared to the NS except in the case of 250 Hz. KEM without applying the VSS was − 0.406 ± 0.218 Nm/kg. KEM at 180 Hz, and 190 Hz was − 0.384 ± 0.243 Nm/kg (Z-value: − 4.462, *p* < 0.001), and − 0.397 ± 0.230 Nm/kg (Z-value: − 2.659, *p* = 0.008), respectively. There was a significant difference between all stimulus conditions. KEM changed from − 0.384 ± 0.243 Nm/kg at 180 Hz to − 0.397 ± 0.230 Nm/kg at 190 Hz (Z-value: − 4.544, *p* < 0.001) and to − 0.404 ± 0.225 at 250 Hz (Z-value: − 4.991, *p* < 0.001). The change between 190 Hz and 250 Hz was significant as well (Z-value: − 3.861, *p* < 0.001).

For KFM_2, when the VSS applied the value decreased significantly. KFM_2 in the case of NS was 0.111 ± 0.048 Nm/kg; 0.080 ± 0.029 at 180 Hz (95% CI: 0.02–0.05, *p* = 0.001); 0.083 ± 0.028 at 190 Hz (95% CI 0.02–0.05, *p* = 0.02) and 0.055 ± 0.023 at 250 Hz (95% CI: 0.04–0.08, *p* < 0.001).

Similar to KEM, there were significant difference between all VSS conditions. Between 180 Hz and 190 Hz KFM_2 changed from 0.080 ± 0.029 Nm/kg to 0.083 ± 0.028 Nm/kg (95% CI: − 0.004- -0.001, *p* = 0.003); between 180 Hz and 250 Hz from 0.080 ± 0.029 Nm/kg to 0.055 ± 0.023 (95% CI: 0.02–0.03, *p* < 0.001) and between 190 Hz and 250 Hz from 0.083 ± 0.028 Nm/kg to 0.055 ± 0.023 (95% CI: 0.02–0.03, *p* < 0.001).

When the VSS applied, AEM increased significantly. Compared to the NS (− 0.465 ± 0.361 Nm/kg), AEM at 180 Hz, 190 Hz, and 250 Hz was − 0.568 ± 0.359 Nm/kg (Z-value: − 4.598, *p* < 0.001), − 0.597 ± 0.361 Nm/kg (Z-value: − 5.261, *p* < 0.001), and − 0.553 ± 0.351 (Z-value: − 4.454, *p* < 0.001), respectively. In addition, there was a significant difference between all frequencies. Between 180 Hz and 190 Hz AEM changed from − 0.568 ± 0.359 Nm/kg to − 0.597 ± 0.361 Nm/kg (Z-value: − 3.689, *p* = 0.015); between 180 Hz and 250 Hz from − 0.568 ± 0.359 Nm/kg to − 0.553 ± 0.351 Nm/kg (Z-value: − 3.486, *p* < 0.001) and between 190 Hz and 250 Hz from − 0.597 ± 0.361 Nm/kg to − 0.553 ± 0.351 Nm/kg (Z-value: − 4.302, *p* < 0.001).

SM without the VSS was 0.446 ± 0.189 Nm/kg. When the VSS of 180 Hz, 190 Hz, and 250 Hz was applied, SM increased to 0.481 ± 0.195 Nm/kg (Z-value: − 5.785, *p* < 0.001), 0.484 ± 0.202 (Z-value: − 5.847, *p* < 0.001), and 0.473 ± 0.195 (Z-value: − 5.354, *p* < 0.001), respectively. In addition, there was a significant difference between all frequencies (180 Hz vs. 190 Hz, Z-value: − 2.643, *p* = 0.008; 180 Hz vs. 250 Hz, Z-value: − 4.916, *p* < 0.001; 190 Hz vs. 250 Hz, Z-value: − 5.696, *p* < 0.001).

HPP_1 increased when applying the VSS of 190 Hz and 250 Hz. HPP_1 for the NS was 0.787 ± 0.532 W/kg. HPP_1 at the 190 Hz and 250 Hz was 0.851 ± 0.595 W/kg (Z-value: − 5.782, *p* < 0.001) and 0.837 ± 0.604 W/kg (Z-value: − 4.846, *p* < 0.001), respectively, which was statistically different from the 180 Hz stimulus. In case of the 180 Hz vs. 190 Hz, HPP_1 was 0.786 ± 0.584 vs. 0.851 ± 0.595 (Z-value: − 4.710, *p* < 0.001) and in case of the 180 Hz vs. 250 Hz was 0.786 ± 0.584 W/kg vs. 0.837 ± 0.604 W/kg (Z-value: − 6.463, *p* < 0.001).

KNP had a statistically significant tendency to decrease. KNP was − 0.170 ± 0.119 W/kg in the case of NS, while at 180 Hz it was − 0.141 ± 0.091 W/kg (Z-value: − 2.688, *p* = 0.007) and − 0.141 ± 0.092 W/kg at 190 Hz (Z-value: − 2.427, *p* = 0.015). There was no significant difference between the 250 Hz stimulus and the NS conditions, whereas the 250 Hz condition showed a significant difference from both the 180 Hz and 190 Hz. In the case of 250 Hz vs. 180 Hz the value was − 0.154 ± 0.102 W/kg vs. -0.141 ± 0.091 W/kg (Z-value: − 3.584, *p* < 0.001), and in the case of 250 Hz vs. 190 Hz the value was − 0.154 ± 0.102 W/kg vs. -0.141 ± 0.092 W/kg (Z-value: − 2.28, *p* = 0.006).

ANP increased in all of stimulus conditions. ANP during the NS was − 0.135 ± 0.090 W/kg, and in the case of 180 Hz, 190 Hz, and 250 Hz stimulation was − 0.214 ± 0.138 (95% CI: 0.05–0.13, *p* < 0.001), − 0.222 ± 0.1137 (95% CI: 0.06–0.11, *p* < 0.001), and − 0.197 ± 0.126 (95% CI: 0.04–0.08, *p* < 0.001), respectively. In addition, there was a significant difference between 190 Hz and 250 Hz (95% CI: − 0.04 - -0.01, *p* = 0.004) stimuli.

## Discussion

The present pilot study has examined the transition from level walking-to-stair ascent of the elderly by comparing with young adults and investigated the effect of the VSS on the transition movement in the elderly and the changes that occur depending on the frequency of the VSS.

Participants in the present study are the same as in our previous study [27]. Our previous study [27] examined the influence of local tendon vibration on postural sway during sit-to-stand movement, whereas the present study investigated the effect of the vibrotactile somatosensory stimulus on neuromotor control of the transition movement from level walking-to-stair ascent. That is, the topic, measure and analysis methods, the analyzed parameters and the motion in each study are different.

During the transition movements, in the first half of the stance, the elderly presented a higher function of the hip extensor to pull up compared to young adults. In the second half of the stance, dorsiflexion and plantar-flexor moment and power to the push-up was higher in the elderly than the young adults. When the VSS was applied, those results were further developed. The hip extensor function for the pull-up and the plantar-flexor function for the push-up increased. Consequentially, the extensor function of the lower extremity (i.e., support moment) increased during the entire stance phase. In addition, joint moment and power during the single-limb support phase were affected by the change in frequency of the VSS.

### Features of the transition from level walking to stair ascent in the elderly

In the early swing phase, compared with young adults, the elderly showed higher dorsiflexion (AA_1), followed by higher hip flexion (HA_1) and dorsiflexion (AA_2). This could be the modality to pass a step of the staircase safely. In other words, it may be a strategy to ensure toe-clearance to prevent tripping over the staircase.

After that, lower dorsiflexion (AA_3) appeared in the elderly compared to young adults. It is possibly being used to clearly land the foot on the staircase through more ankle extension than the young adults. Consequently, ankle extension induces smooth foot landing, resulting in a decreasing impact force from the step during the weight acceptance.

In the stance phase, both groups used dominantly the extensor of the hip and knee joint to the lift body. However, the modality of use was opposite between the two groups. The elderly predominantly used hip extensor, whereas the young adults used the knee extensor. This can be attributed to weakness of the quadriceps femoris. It is a well-known fact that the elderly have lower muscle strength in the lower extremity compared with young adults. In particular, Hortobágyi et al. [37] reported that older adults had 60% lower maximal leg press moments compared with young adults. Due to this, the support moment could appear low in the elderly (SM_1). This could induce more activity of the hip extensor to compensate for the weakened knee extensor, and in the case of the plantar-flexor, it contributes to some compensation. These features could provide a clinical reference for training the quadricep muscles in the elderly.

Although joints of the hip and knee extended continuously in both groups, dorsiflexion decreased by plantar-flexor activity (AP_1) in young adults, whereas it was sustained in the elderly. This may be a strategy of the elderly to ensure stability while ascending the stairs. First, sway of the shank can be minimized by fixing the ankle joint. Second, if the ankle joint is extended, the position of the center of mass (COM) increases, which may cause postural instability due to COM acceleration. Finally, knee extension that is created by pulling the shank back, which contributes to the forward acceleration of the COM, can be prevented by restricting activity of the plantar-flexor.

In the late stance phase, dorsiflexion and knee flexion were higher in the elderly than in the young adults. This can contribute to a reduction in COM height, foot landing of the contralateral leg, and weight shift to the opposite leg. For this, the support moment (SM_3) increased more, which was attributed to the extensor moments of the hip and knee joints. This is a modality for securing energy for the subsequent push-up and compensating for the weakened quadriceps femoris in the opposite leg.

At the end of the stance phase, the plantar-flexor positive power is greater in the elderly. This was attributed to the larger dorsiflexion just earlier, and was a strategy to counter the more flexed dorsiflexion and assist the pull-up function of the proximal extensor of the opposite leg.

In summary, the elderly tended to use more hip extensors than young adults to lift the body, and to presumably ensure stability during locomotion, the extension of the ankle joint was restricted. In addition, the plantar-flexor power increased by increasing the flexion of the distal segments, possibly to assist the pull-up function of the opposite leg.

### Effect of VSS on the transition from level walking to stair ascent in the elderly

In the swing phase, after toe-off, dorsiflexion (i.e., AA_1) increased, followed by a decrease in knee joint flexion. Toe clearance was ensured more by the increased dorsiflexion, and the decreased knee joint flexion could have contributed to control foot-landing by compensating for increased dorsiflexion. Consequently, the VSS induced further toe clearance, resulting in more stable stepping and more cautious foot contact.

In the stance phase, the moments and positive powers of the hip extensor increased. This could indicate that the function of the hip extensor to lift-up the body was enhanced by the VSS and that features of the elderly in the pull-up phase were also developed. In addition, the support moments increased. Hence, the results indicated that VSS increased function of the body support during the single-limb support phase and contributed to the pull-up of the body by activating the hip extensor. Furthermore, moments of the plantar-flexor increased slightly during the first half of the stance phase, which may have contributed to an increase in the support moments. Consequently, VSS contributed to an increase in the body support function while transitioning from the double limb support stance phase to the single-limb support stance.

In the second half of the stance phase, an increase in the knee joint flexion, dorsiflexion, and support moments appeared; that is, the VSS possibly further reinforced the body support (i.e., an increase in the SMs and negative plantar-flexor powers) and contributed to increasing the push-up of the plantar-flexor and assisting the pull-up of the contralateral limb.

These results indicated that the VSS could further develop characteristics of the transition movement in the elderly. In summary as follows:

The VSS possibly ensures safer toe clearance by increasing dorsiflexion, enhancing lift-up function of the hip extensor, increasing body support during the single-limb support phase, and assisting in the push-up of the plantar-flexor and the pull-up of the contralateral limb.

Despite the listed effects of VSS, the duration of the transition can be lengthened due to the increase in joint flexion and support moment. With increasing joint flexion, more extensor activity and antagonist muscle activation are required to prevent lower-limb collapse, and to restrict joint movement, respectively. These are factors that increase the joint stiffness. Thus, future studies should include spatiotemporal variables and electromyography (EMG).

### Parameters depending on the frequencies of the VSS

For the hip joint angle, although there were no significant differences under non-stimulus and stimulus conditions, HA_2 had a tendency to increase. For the knee joint angle, there was a significant difference between non-stimulus and stimulus in KA_1 and KA_3, despite no difference between frequencies. For the ankle joint angle, there was a statistically significant difference between non-stimulus and stimulus in the AA_1 and AA_4. In particular, statistical significance was found between 180 Hz and 250 Hz for AA_4.

Overall, the VSS can be considered to cause a change in kinematics, and the trend seems to be common at all frequencies. This common trend may have resulted from the fixed dimensions of the staircase. However, differences depending on frequencies may occur in kinetic parameters considering that the joint angles of each stimulus condition were different.

As shown in Table 2, statistically significant differences between the non-stimulus and stimuli appeared between each frequency pair. Furthermore, significant frequency-dependent differences were found in the support moment, a sum of extensor moments of the lower limb joints [36, 38], as shown in Table 4. Steyvers et al. [39] measured motor evoked potential (MEP) depending on vibration frequency and revealed that frequency-dependent effect appeared in the MEPs. Therefore, it is indicated that the VSS affects joint kinetics while transitioning from level walking to stair ascent, and thus it can be presumed that joint kinetics can be regulated depending on the VSS frequency; that is, the VSS could influence neuromotor control (NMC). As a result, performance degree in sub-biomechanical tasks (i.e., weight acceptance, pull-up, body support, and push-up) during transitioning from flat ground walking to stair climbing will be affected.

There were significant differences between each frequency pair in the KNP for foot landing, the HPP_1 for the pull-up, the SM for preventing lower-limb collapse, and the ANP for ensuring stability, supporting the lower-limb and assisting the opposite leg. Although not all frequencies, this indicated that the same function is performed during the transition, but the degree varies with the vibration frequency; that is, it suggests that the NMC of the transition is frequency dependent.

In conclusion, the VSS has the possibility to control the degree of performance of sub-biomechanical tasks by affecting the NMC. It can further develop the characteristics of the transition movement in the elderly. For more insight, future investigations on EMG and muscle synergy are required.

### Role of VSS in assisting level walking-to-stair ascent and its application

In this study, it was found that the elders’ characteristics of the transition motion differed from those of young adults. The predominant characteristics were larger hip joint flexion (HA_1), sustained dorsiflexion and larger dorsiflexion (AA_4), higher moment (HM_2) and power (HP_2) of the hip extensor, higher power of the plantar-flexor (AP_3) and the lower support moment (SM_1). These characteristics of the elderly might be an adaptation of motor control strategy according to neuro-physiological changes due to aging.

When the VSS was applied, some characteristics (i.e. AA_4, HM_2, HP_2, and SM_1) and unexpected parameters (i.e. AM_2, AM_3, AP_2, SM_2, and SM_3) were affected; that is, the function of the hip extensor in the first half of the stance phase, function of the plantar-flexor in the second half of the stance phase, and support moment in the entire stance phase. Consequently, the VSS enhanced motor control of the transition movement in the elderly, and motor control can be regulated depending on the frequency of the VSS as shown in Tables 1, 2, 3 and 4.

These findings suggest that the VSS can be utilized as a means of assisting the transition movement in the elderly. To apply the VSS, various commercial linear actuators can be used. The linear actuator used in the present study was small. Its diameter and thickness were 9 mm and 3.4 mm, respectively. In addition, to detect the transition movement or any motions, an inertial measurement unit (IMU) device and a device combined with an accelerometer and gyroscope can be used.

There is already a variety of sensors and methodology to detect and recognize human movement. In other words, if the VSS, IMU, power supply, communication module, and regulator for voltage and frequency are combined and miniaturized, the assistance device can be realized. Park et al. [40] and So et al. [41] used inertial sensors to detect gait events and small vibrators to apply somatosensory stimulation, and suggested an algorithm for detecting and stimulating. In addition, portable devices that detect motion and apply stimulation have been used in various clinical studies [42–44]. Therefore, the results of this study can be sufficiently utilized as clinical reference to assist the elderly in their movements, and support the design of assistive devices to enhance motor control by combining the above-mentioned devices and methodologies.

### Limitations

This pilot study has some limitations. 1) the number of participants in this study was small and the sample size between both groups did not the same. 2) the steady-state stair ascent was not investigated in this study. That is, further insight into elders’ stair walking, it is required that more sample size and comparing the transition from level walking-to-stair ascending with the steady-state stair ascent.

## Conclusion

The present pilot study showcased the features of the transition from level walking-to-stair ascent in elderly people, and that the VSS may accentuate the features of the transition movement of the elderly. Additionally, it indicated that the frequency of the VSS has the potential to regulate neuromotor controls of the transition.

The results of this study can provide information on the transition movement of the elderly and the references for clinical rehabilitation training to assist and improve their movements. In addition, the observations in this pilot study should be considered when planning future research in the development of motion assistance devices.

## Data Availability

The datasets used and/or analyzed during the current study are available from the corresponding author on reasonable request.

## Abbreviations

VSS: Vibrotactile somatosensory stimulus
HA: Hip joint angle
KA: Knee joint angle
AA: Ankle joint angle
HM: Hip joint moment
KM: Knee joint moment
AM: Ankle joint moment
HP: Hip joint power
KP: Knee joint power
AP: Ankle joint power
SM: Support moment
HFM: Hip flexor moment
HEM: Hip extensor moment
KFM: Knee flexor moment
KEM: Knee extensor moment
AEM: Ankle extensor moment
COM: Center of mass
EMG: Electromyography
MEP: Motor evoked potential
